# The London Hospital: An Immediate Need

**Published:** 1916-08-05

**Authors:** 

**Affiliations:** London Hospital, E.


### THE EDITOR'S LETTER-BOX.

### The London Hospital: An Immediate Need.

The following appeared in the *Times* of July 29:

Sir,—On my return to work after a prolonged absence owing to an accident, I met with a report which makes me almost wish that the motor which knocked me over had completed its Juggernaut task more effectually.

"The London Hospital," says the report "must either do less work or lower the standard of its work." During my absence, alarmed at the financial condition of the London Hospital, a committee of business men, aided by experts, have gone very carefully into the expenditure of every one of its many departments. The reference to them was to report upon any economies which could be made. "We find," they report, "that every department has already reached a point beyond which no further economy can be made. Buying is carried out with every possible thought and care; consumption is checked and counter-checked in a way that repeatedly aroused our admiration." This is very satisfactory, as I know how loyally Mr. Morris, the house governor, and Miss Luckes, the matron, and the heads of the various departments have striven to reach this point. And then the report goes on: "The impression which has been forced upon us is that the only possibility of reducing expenditure is to lower the standard of the work or to lessen the amount of work attempted."

I ask a public who have always helped the London Hospital generously for the last 140 years whether either of these alternative suggestions are to be carried out? Are we to lessen the amount of work done for the East End sick poor at a moment when they need our help as much as, if not more than, ever? Are they to tell their husbands, sons, and brothers fighting at the Front that those left at home cannot be cared for in sickness any more? Are we to tell mothers that it is useless to bring their children to us because we have no money to help them back to health?

The year before Waterloo the *Times*, on Thursday, August 4, 1814, wrote as follows:

"While the sympathies of the British people have been excited in favour of the sufferers of other nations, our own most excellent charitable institutions must not be suffered to languish under incompetent funds, by which they are of necessity obliged to withhold their Samaritan benefit. We are glad to see that the merchants, bankers, and public bodies have promptly come forward with liberal donations in behalf of the London Hospital, the funds of which had, indeed, for the last few years, been found greatly inadequate to afford the relief required by the increasing demands of an extended population. It is, in fact, the only hospital in the eastern district, and may almost be termed the Provincial Hospital for the County of Essex."

Surely the same applies with greater force to-day, when the numbers of those who are fighting and the numbers left at home are one-hundred-fold greater, and the condition of the hospital far more serious.

Take the other alternative. Is the standard of work at England's largest hospital to be lowered? If so, would it ever get back to its high level? Are we to teach young doctors and nurses that second-rate work will do? Ought the standard of work for the sick, of all work, to be lowered? Of course it can be lowered; anyone can do bad work, but it shall not be until this miserable policy is forced upon us. Having probably owed my life to the perfection of doctoring, nursing, and care that I have lately received, perhaps I am realising more acutely than ever what it would mean to lower the standard—in other words, not to do the best possible for the sick and injured. To do less well for these people than we know to be possible means the death of many of them. There is no getting over this. So there, stated frankly, and without any exaggeration, is the serious position in which this great hospital finds itself to-day. Is it to go under, not because it has done its work badly all these years, but because it has done it well and to the full measure of its responsibilities? The War Office and the Admiralty would, I am sure, gladly testify to the help we have given by taking in soldiers (nearly 4,000) and in providing 541 doctors and 150 nurses, and in helping in many other ways when called on to do so.

I am told that it is hopeless to issue an appeal in these days for other than war work. But I cannot help trying to avert the calamity of reducing our work, or the disgrace of doing it badly, by making this earnest appeal for help. We are not in debt, so every penny given will go to the present and future sick, and not to pay back bills.—Yours faithfully, Knutsford.

London Hospital, E., July 26.

P.S.—To give some idea of the actual work done, we have sixty operations under anæsthetics every day of the year. Out of 20,000 operations last year we had only two deaths under anæsthetics. We treat every year about 18,000 in-patients and 179,000 out-patients.

